# Complete Genome Sequence of a γ-Hexachlorocyclohexane Degrader, *Sphingobium* sp. Strain TKS, Isolated from a γ-Hexachlorocyclohexane-Degrading Microbial Community

**DOI:** 10.1128/genomeA.00247-16

**Published:** 2016-04-07

**Authors:** Michro Tabata, Satoshi Ohhata, Toru Kawasumi, Yuki Nikawadori, Kouhei Kishida, Takuya Sato, Yoshiyuki Ohtsubo, Masataka Tsuda, Yuji Nagata

**Affiliations:** Graduate School of Life Sciences, Tohoku University, Sendai, Japan

## Abstract

Here, we report the complete genome sequence of a γ-hexachlorocyclohexane (γ-HCH) degrader, *Sphingobium* sp. strain TKS, which was isolated from a γ-HCH-degrading microbial community. The genome of TKS consists of two chromosomes and nine plasmids. The *lin* genes for conversion of γ-HCH to β-ketoadipate are dispersed on chromosome 1 and three out of the nine plasmids.

## GENOME ANNOUNCEMENT

γ-Hexachlorocylohexane (γ-HCH; also called γ-BHC or lindane) is a chlorinated organic insecticide that has caused serious environmental problems due to its toxicity and long persistence in upland soils (1, 2). Our γ-HCH-enriched liquid cultivation of a microbial community from a sediment from Kyushu Island, Japan, contaminated with HCH isomers, used 1/10W minimal medium (3) containing γ-HCH (50 µg/ml) as a sole carbon source. Subsequent repeated single-colony isolation processes used 1/10W minimal solid (1.5% agar) medium containing γ-HCH (750 µg/ml), which led to the isolation of a γ-HCH-degrading strain, *Sphingobium* sp. TKS, and a non-γ-HCH-degrading strain, *Pseudomonas* sp. TKP. The complete genome sequence of the latter strain TKP was previously determined (4). In this study, we determined the complete genome sequence of the former strain, which has been deposited in the Japan Collection of Microorganisms (JCM) under the accession number JCM 19687.

The TKS genome was sequenced using 454 GS-FLX+ (Roche) system and the HiSeq 2000 (Illumina) mate-pair sequencing system, which was operated by Eurofins Genomics Inc., and 625,334 reads and 7,309,649 reads, respectively, were obtained. These reads were assembled using Newbler (Roche) to generate initial draft sequence data consisting of 33 scaffolds and 917 contigs. The finishing was facilitated using GenoFinisher and AceFileViewer (5). The complete genome sequence was annotated by the NCBI Prokaryotic Genome Automatic Annotation Pipeline (PGAAP), and the resulting annotation was subjected to manual curation using the annotation support tool of GenomeMatcher (6). By referencing annotation data obtained from the Microbial Genome Annotation Pipeline (http://www.migap.org), we corrected the start codon positions and added several genes that were missing in the PGAAP annotation.

The TKS genome consists of two circular chromosomes, Chr1 (4,249,857 bp, 63.4 % G+C, 4,173 open reading frames [ORFs]) and Chr2 (989,120 bp, 62.9% G+C, 844 ORFs), and nine circular plasmids, pTK1 (520,614 bp, 62.8% G+C, 470 ORFs), pTK2 (195,308 bp, 59.8% G+C, 182 ORFs), pTK3 (87,635 bp, 61.7% G+C, 92 ORFs), pTK4 (75,938 bp, 62.0% G+C, 86 ORFs), pTK5 (53,908 bp, 60.9% G+C, 73 ORFs), pTK6 (34,300 bp, 62.8% G+C, 35 ORFs), pTK7 (9,585 bp, 60.3% G+C, 12 ORFs), pTK8 (7,223 bp, 59.7% G+C, 11 ORFs), and pTK9 (5,391 bp, 60.9% G+C, 8 ORFs). Two chromosomes carry three copies of rRNA operons and 54 tRNA genes. The TKS-specified *linA*, *linB*, and *linC* genes, and *linRED* cluster for conversion of γ-HCH to maleylacetate (7) are almost identical to those from an archetypal γ-HCH-degrading strain, *S. japonicum* UT26 (8), and are dispersed on Chr1 (*linB* and *linC*), pTK3 (*linB* and *linC*), pTK4 (*linA* and *linC*), and pTK6 (*linDER* cluster). The UT26-specified maleylacetate reductase (MAR) gene (*linF*_UT26_) for conversion of maleylacetate to β-ketoadipate (9) was not found in the TKS genome. Chr1 in TKS instead carries *linFb*, whose product is 49% identical to LinF_UT26_ at the amino acid level, and the expected MAR activity of LinFb has been experimentally confirmed (Tabata et al., unpublished data).

### Nucleotide sequence accession numbers.

Sequences with annotation information have been deposited in GenBank under the accession numbers CP005083, CP005084, CP005085, CP005086, CP005087, CP005088, CP005089, CP005090, CP005091, CP005092, and CP005093 for Chr1, Chr2, pTK1, pTK2, pTK3, pTK4, pTK5, pTK6, pTK7, pTK8, and pTK9, respectively.
